# Avoidance, escape and microstructural adaptations of the tea green leafhopper to water droplets

**DOI:** 10.1038/srep37026

**Published:** 2016-11-15

**Authors:** Meizhen Lin, Liette Vasseur, Guang Yang, Geoff M. Gurr, Minsheng You

**Affiliations:** 1 Institute of Applied Ecology, Fujian Agriculture and Forestry University, Fuzhou 350002, China; 2 Fujian-Taiwan Joint Centre for Ecological Control of Crop Pests, Fujian Agriculture and Forestry University, Fuzhou 350002, China; 3 Key Laboratory of Integrated Pest Management for Fujian-Taiwan Crops, Ministry of Agriculture, Fuzhou 350002, China; 4 Department of Biological Sciences, Brock University, 1812 Sir Isaac Brock Way, St. Catharines, ON L2S 3A1, Canada; 5 Graham Centre, Charles Sturt University, Orange, NSW 2800, Australia

## Abstract

Rain and dew droplets can dislodge or drown small insects and may be important factors that drive adaptations for avoidance and escape. Studying the microstructure of small insects and insect behaviour can help understand these adaptations. We quantified avoidance behaviour and entrapment of nymphs and adults of the tea green leafhopper (*Empoasca onukii*) using simulated rainfall onto host plant foliage and made observations of pretarsi and abdomen microstructures. Adults responded rapidly to simulated rainfall and escaped by jumping whilst most young nymphs were washed from water-sprayed leaves though older nymphs tended to remain on leaves and subsequently escaped from water droplets. Adults had denser covering of water-repelling brochosomes on pretarsi and abdomen surface than nymphs, and were able to stand on water film whilst most nymphs had multiple penetrating tarsi. Removal of brochosomes from the abdomen of adults reduced hydrophobicity, demonstrating the hydrophobic significance of brochosomes in the capacity of leafhopper to escape from water droplets. Nymphs exhibited a higher pull-off force than adults. This research is one of the few studies to focus on the wettability and water avoidance of small insect pests and has implications for pest management.

Many organisms, including invertebrates, have adaptations such as hydrophobic surfaces to contend with rainfall and dew. Such properties have interested researchers for the development of biomimetic structures and materials^1^,^2^,^3^,^4^. Most small invertebrates have difficulty staying on plants in a storm due to wind and water action. For example, simulated rain can wash apterous *Sitobion avenae* Fabricius^5^ or mites *Aeaphylla theae* Watt^6^ from plants. Dew and rainfall may cause mortality of small insects by entrapment and dislodgement from foliage and this is expected to drive microstructural and behavioural adaptations. Simulated rainfall can wash aphids from plants and their populations are significantly suppressed both in the field and under laboratory conditions^7^,^8^.

Simulated rain can lead to aphid movement between plants, thus promoting dispersal and plant occupancy^9^. This demands that insects can, after water-induced dislodgement, escape from droplets before drowning. Much more generally among insect, however, non-wetting surfaces are important for interacting with environmental water. For example, a lacewing can take off from a water surface even though its wings contact with the water as a result of arrays of macro-trichia that project from the veins and a dense nano-netting beneath the hairs on the cuticle surface reduces wetting of the wings^10^. The superhydrophobic cuticle of termites is the result of nano-micrasters and macrotrichia on the wing membrane and can allow them to escape from puddles^11^.

The leafhoppers are reported to coat their integuments with brochosomes after moulting^12^. Only some lineages of nymphs may produce brochosomes^12^,^13^. Brochosomes are spherical microparticles with a honeycomb-like structure that are composed of proteins, possibly with additional components^14^. Brochosomes are excreted from the hindgut and applied to the integument by grooming. Once the liquid brochosomes have dried, leafhoppers begin repeated bouts of grooming to spread brochosomes over their body^15^,^16^. Dong and Huang^16^ observe that leafhopper individuals synchronously groom when on leaves with water droplets and propose that grooming behaviour is related to the presence of water. Brochosomes protect the integument by repelling water and sticky insect excrement^15^,^17^. Rakitov and Gorb^15^ report that brochosomes turn the leafhopper wings into a surperhydrophobic state with the average water contact angles (CAs) of 165∼172°. In this study, we aimed to test whether experimental removal of brochosomes affected the hydrophobicity of leafhopper body parts and thereby determine their significance to the capacity of the live insect to avoid wetting and escape from water.

We choose the tea green leafhopper, *Empoasca onukii* Matsuda (Cicadellidae: Hemiptera), (previously named *Empoasca vitis* (Göthe) in China) as our experimental species, which is a significant insect pest in Asian tea plantations. It has a small body length with males typically *ca*. 2.5 mm and females *ca.* 3.0 mm^18^. Both nymphs and adults are phloem feeders on the tender tea bush shoots, where females oviposit. Therefore all the life stages of the species impact on this economically valuable plant. It is known that rain and wind disturbance can interfere with the population dynamics of *E. onukii,* increasing nymph mortality^19^,^20^ to the extent that the number of rainy days in March and April can be used to predict the first population peak of *E. onukii*^19^. It is suggested that both *E. onukii* adults and nymphs cannot move when there is dew on tea leaves^21^, however this has yet to be tested. We speculated that the hydrophobicity of the insect, particularly the pretarsus would be significant in their capacity to escape from water droplets.

*Empoasca onukii* is known for its capacity to move rapidly. The adults can leap and fly, and the nymphs run quickly along the tea stem when disturbed. Both adults and nymphs can hide under leaves. It is thought that the impact of insecticide applications for this pest is reduced because of this capacity to move rapidly and seek refuge^22^,^23^. Potentially, the adaptations to avoid dislodgement by rainfall, and wetting by water films and droplets could also confer an advantage by reducing exposure to insecticide. We speculated that the escape behaviours might be different between *E. onukii* adults and nymphs, with adults having the capacity to fly away with their wings and the presence of brochosomes^15^. Whether the nymphs are superhydrophobic had yet to be examined, especially in terms of presence of brochosomes or any micro- and nanoscale hierarchical surfaces.

In this study, we assessed the behavioural responses of *E. onukii* nymphs and adults to simulated rainfall and examined the surface microstructures of the insect body parts that have direct contact with water at different life stages by scanning electron microscopy. To test the role of brochosomes, we removed them by using polyvinylsiloxane, and conducted experiments to compare the pull-off force and contact angle between nymphs and adults and water droplets both with and without the brochosomes. Rakitov and Gorb have reported that the hydrophobicity is reduced when brochosomes are removed from the wings of leafhoppers^15^. We therefore hypothesized that brochosomes found on body parts other than the wings might play an important role in hydrophobicity of the insect.

## Results

### Escape behaviour

**Figure 1:**
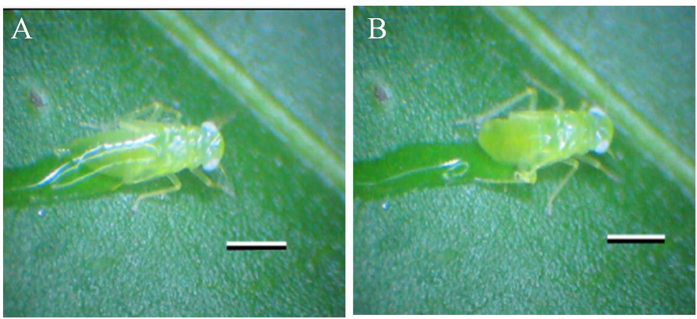
Escape behaviour of the 5^th^ instar *E. onukii* nymph from a simulated raindrop on the abaxial surface of a tea leaf. (**A**) Entrapment of the nymph in simulated raindrop; and (**B**) Crawling away of the nymph from the simulated raindrop. Scale bars: 1 mm.

Response time of adult *E. onukii* was 0.030 ± 0.005 s from the arrival of the first droplet on the leaf. Although some water came into contact with some adults, all escaped successfully by jumping (see Table 1 and supplementary material, Video S1). At the other extreme, no first instar nymphs escaped wetting, all became trapped in the resulting water film, and were washed from the leaf (see supplementary material, Video S2). For N2-3 nymphs, only 15% of individuals escaped the water droplets with a mean escape time of 48.70 ± 3.05 s after cessation of spraying for 1 min (Table 1). It was difficult for the nymphs to remove their abdomen from the water droplets (see Fig. 1 and supplementary material, Video S3). Though able to draw their anterior portion from the water, adhesion between the water and the abdomen and hind legs often prevented escape. For the larger N4-5 nymphs, 85% of the individuals escaped droplets and did so at a mean of 17.40 ± 2.78 s after cessation of spraying for 1 min i.e., less than half the time taken by N2-3 nymphs (Table 1).

### Microstructure of the pretarsi and abdomen

#### Microstructure of pretarsi

**Figure 2:**
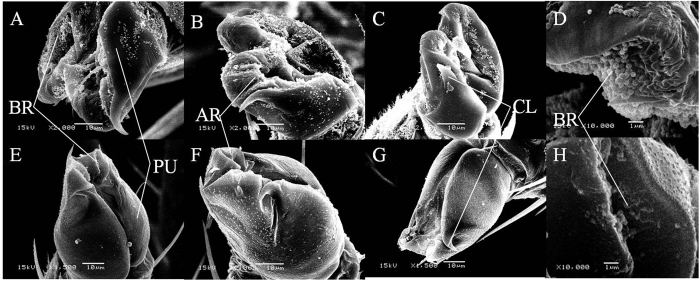
SEM images of pretarsus of *Empoasca onukii*. (**A**) foreleg of adult; (**B**) middle leg of adult; (**C**) hind leg of adult; (**D**) surface of the arolium in foreleg pretarsus of adult; (**E**) foreleg of nymph; (**F**) middle leg of nymph; (**G**) hind leg of nymph; and (**H**) surface of the arolium in foreleg pretarsus of nymph. (BR) brochosomes; (PU) pulvillus; (AR) arolium; (CL) claw.

SEM revealed that the shape of adult pretarsi was flatter than nymph pretarsi. Adult pretarsi had a pair of wide flat arolia (terminology according to Snodgrass^24^) between the claws of each pretarsus (Fig. 2A,B,C). On the apex of the arolium, there were many fine grooves, which were filled with numerous brochosomes (Fig. 2D). The basal part of the pretarsus, which was about a third the length of the pretarsus, consisted of a pair of pulvilli. Arolia and pulvilli were fused with no demarcation. For N4-5 nymphs, the pretarsus was more elongated, with a pair of large, pouch-like pulvilli that were nearly three-quarter of the pretarsus length. The arolia between the nymph claws (Fig. 2E,F,G) had a more marked concavity than those of the adult. Few brochosomes were present on nymph pretarsi (Fig. 2H).

#### Microstructure of the abdomen

**Figure 3:**
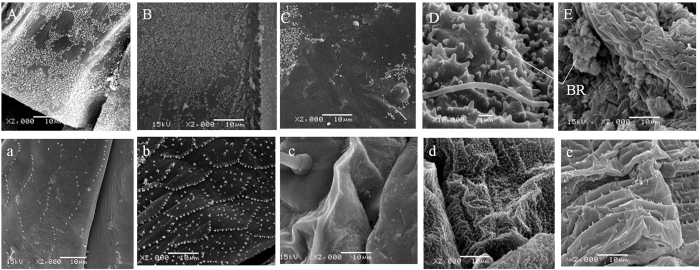
SEM images of the *E. onukii* abdomen. (**A–E**) are images of abdomen cuticle with intact integuments, while (**a**–**e**) are images of abdomen cuticle with artificially bared integument. (**A**) and (**a**) represent adult dorsal abdomen; (**B**) and (**b**) represent the adult ventral abdomen; (**C**) and (**c**) represent nymph (1st to 5th) dorsal abdomen; (**D**) and (**d**) represent the ventral abdomen of younger nymph (1st and 2nd); (**E**) and (**e**) represent the ventral abdomen of older nymph (3rd to 5th); BR represent brochosomes. Scale bars: 1 μm for (**D**) and 10 μm for others. In (**D**) the size of brochosomes are similar to the micropapillae, and in (**E**) brochosmomes on the ventral abdomen of the nymph are clumped and have not been spread over its body.

For adults, masses of brochosomes were distributed on both dorsal and ventral surfaces of the abdomen (Fig. 3A,B). For nymphs, brochosomes were mainly located on the ventral surface of the abdomen and the number of brochosomes increased with the instar (Fig. 3D,E), while only few brochosomes on the dorsal surfaces (Fig. 3C). The average diameter of brochosomes was 0.30 ± 0.01 μm for both nymphs and adults. When brochosomes were removed, both dorsal and ventral aspects of the adult abdomen had uniform fish-scale structures formed with microtrichia (Fig. 3a,b). The microtrichia on the ventral abdomen (0.7–1.6 μm) (Fig. 3b) were slightly longer than those on the dorsal abdomen (0.3–0.6 μm) (Fig. 3a). In all the nymph stages, six lines of sensory setae (45.0–85.0 μm) were distributed on the dorsal surface of the abdomen, and the cuticle was quite glossy (Fig. 3c). The microstructures on the nymph ventral abdomen varied with life stages. For younger instars (1st and 2nd), the ventral surface contained many micropapillae (0.3–0.8 μm) with few brochosomes (Fig. 3D,d) while in older instars (3rd to 5th), micropapillae in the middle part of the ventral abdomen were absent and only a few microtrichia (0.7–1.6 μm) were found (Fig. 3e) but with many brochosomes (Fig. 3E).

### Properties of pretarsi and abdomen

#### Wettability of pretarsi

When individual insects were deposited on water, adults stood on the water surface, with only their pretarsi touching the water surface. Only two adults had their two middle legs penetrating the water surface. In contrast, 17 of the 20 nymphs had at least five legs dipping into the water and three nymphs had all of their legs dipping into the water. The abdomen of all of the nymphs touched the water surface. All insects drowned in the soybean oil because of reduced surface tension.

#### Contact angle measurements of abdomen

**Figure 4:**
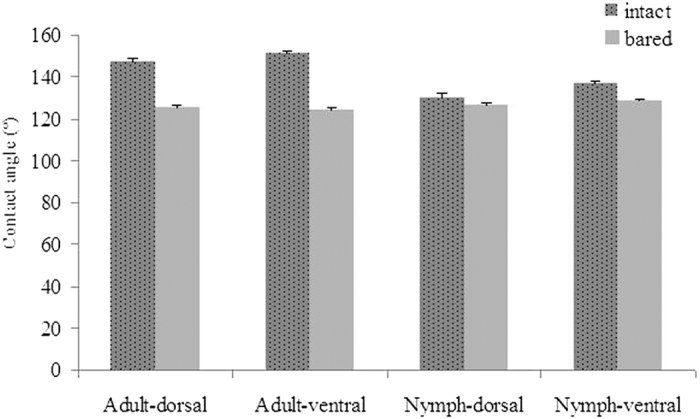
Contact angle values of water on the surface of the *E. onukii* adult and old nymph abdomen (ventral and dorsal) with or without the presence of brochosomes. Intact bars (dark grey with dots): leafhopper integument with brochosomes; bared bars (pale gray): leafhopper integument without brochosomes. Error bars indicate standard deviation.

For adults, the CAs of ventral and dorsal abdomen surfaces were significantly reduced from an average of 149.10 ± 1.03° to 125.35 ± 0.69° when brochosomes were removed (*t* = 19.812, df = 7, *P* < 0.001) (Fig. 4). A similar reduction was observed for the ventral surface (*t* = 4.001, df = 7, *P* = 0.016) of the nymph abdomen but not for the dorsal surface (*t* = 1.483, df = 7, *P* = 0.212). The nymph abdomen surfaces could be considered hydrophobic, since the average CA was 133.3 ± 1.27° for ventral and dorsal abdomen surfaces with brochosomes, and the average CA was 127.70 ± 0.71° for the bared abdomen surfaces.

#### Pull-off force (POF) of pretarsi

**Figure 5:**
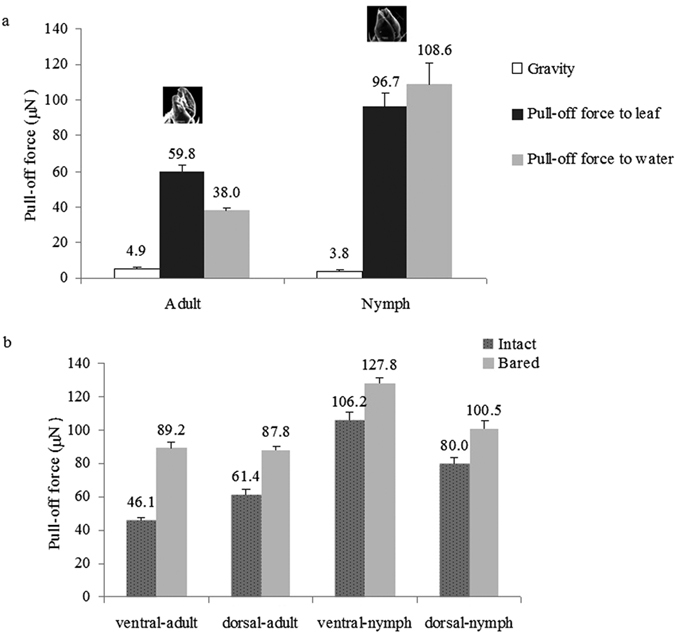
The pull-off force of pretarsus and abdomen. (**a**) Comparison of the pull-off (black bars for leaf interface and gray bars for water interface) and gravity (white bars) forces generated at the insect pretarsi and tea leaf interfaces as well as at the insect pretarsi and water interfaces for adults versus nymphs of *E. onukii*; and (**b**) Comparison the pull-off force between the intact integument (dark grey bars with dots) and artificially bared integument (pale gray bars) forces generated at the insect abdomen-water interfaces for adults versus nymphs of *E. onukii*. Error bars indicate standard deviation.

The POF of nymph pretarsi was significantly greater than that of adults (*P *< 0.0001) (Fig. 5A) whether on tea leaf or water droplet. The POF of nymph pretarsi from water was 108.6 ± 11.9 μN, nearly 28 times its gravitational weight (3.8 ± 0.1 μN), and was higher than the POF from a tea leaf surface (96.7 ± 6.9 μN) (*t* = 3.452, df = 7, *P *= 0.026). For adults, pull-off force of pretarsi from water was significantly lower than the value for tea leaf surfaces (*t* = 7.410, df = 7, *P* = 0.002), representing about 7 times the insect’s gravitational weight (4.9 ± 0.11 μN).

#### Pull-off force of abdomen

For both adults and 4-5th instar nymphs, POF values of brochosome-free abdomens (bared groups) were significantly higher than the values of brochosome-covered integument (intact groups) (*P *< 0.001, Fig. 5B). The difference was especially important for adult ventral abdomen surfaces where the POF value of bared integument (89.2 ± 3.6 μN) was nearly twice as the value of intact integument (46.1 ± 1.2 μN).

With or without brochosomes, the POF values of nymph abdominal surfaces were obviously higher than adults (*P *< 0.001, Fig. 5B). Comparing intact groups, the POF values of adult dorsal side was higher than adult ventral side, but the nymph’s values were opposite, the value of dorsal-nymph was lower than ventral-nymph. When the brochosomes were removed by PVS film, POF of the nymph’s ventral abdomen (127.8 ± 3.3 μN) from a water droplet was significantly greater than the nymph’s dorsal abdomen surface (100.5 ± 5 μN) (*t* = 3.470, df = 7, *P* = 0.026), while the abdominal surfaces of adults did not differ (*t* = 0.239, df = 7, *P* = 0.823), with POF of adult ventral abdomen 89.2 ± 3.6 μN and POF of dorsal side 87.8 ± 2.4 μN.

## Discussion

The present study showed that tea green leafhopper has a range of behavioural and microstructural adaptations, with brochosomes playing a key role in hydrophobicity, to minimise entrapment in water. Adult *E. onukii* can escape entrapment by taking-off rapidly from sites subject to simulated rainfall. Nymphs, in contrast, did not escape from sprayed leaves. Most early instar individuals were washed away in spray run-off. In contrast, older nymphs were able to adhere to the leaf and subsequently escape the water surface tension after cessation of spraying. Our study demonstrates that the adults and nymphs of different developmental stages have different behavioural responses to simulated rainfall but further work is clearly necessary to determine the field significance of this behaviour and the fate of insects of each category.

Under natural conditions, adults would likely find refuge under leaves or within the canopy. In the present experiment, adults left sprayed leaves in an average of 0.03 s after the first droplets hit the substrate (or insect body), a value close to the 0.037 ± 0.006 s that Brackenbury^25^ reported as time for wings to be opened, representing a very rapid response to the spraying. Accordingly, all the adults escaped entrapment and subsequent dislodgement by ‘wash-off’ from the sprayed leaves.

We found that pretarsus microstructure of *E. onukii* varied with life stages. The nymph’s pretarsi resemble those of adult potato leafhopper *E. fabae*^26^, with a concave arolia structure that may assist adhesion to dry leaf surfaces. In contrast, adults of *E. onukii* would more easily re-settle on leaves after dislodgement and have less need of a high pull-off force from dry leaves. Their pretarsal microstructure may constitute a trade-off for greater hydrophobicity and capacity for rapid locomotion from danger. In particular, the pretarsi of adults had more brochosomes than did pretarsi of nymphs and this was reflected in measurement of pull-off force from water droplets. This is consistent with the observation that adults responded to simulated rainfall by very rapid locomotion from the leaf. A low pull-off force of adult pretarsi would assist this escape response, after which the flying adult could – more readily than the wingless nymph^27^ – relocate on host plant tissue and recommence feeding. Hydrophobicity of the adult pretarsus was evident in the observation that this life stage was better able to stand on a water surface without tarsi penetrating the droplet or other body parts contacting the water. This brochosome-associated hydrophobicity of the adult pretarsus is functionally analogous to the water-repellent legs covered with minute hairs with fine nanogrooves of water striders (Gerridae)^4^ and crane flies (Tipulidae) with fine hair structure on their legs^28^.

The nymph pretarsus had a high pull-off force from dry leaves, close to double that of the adult and this may reflect a life-stage specific adaptation to reduce dislodgement in non-flying nymphs that would otherwise lead to reduced feeding activity and increase exposure to soil-associated predators after dislodgement from the foliage^29^. The risks associated with dislodgement are particularly great for less mobile insects such as the wingless form of aphids^27^ and the early instar nymphs of *E. onukii*. Nymphs had fewer brochosomes on pretarsi than the adults and this was reflected in higher pull-off forces from water droplets and the greater extent to which pretarsi penetrated water droplets. Simulated rainfall resulted in most nymphs being trapped in water droplets from which escape was particularly difficult for young nymphs.

Brochosomes appear key in reducing the adhesive force between adult abdomen and water. When brochosomes were removed, POF from water was increased significantly. Rakitov and Gorb^15^ suggest that leafhoppers have evolved brochosomes rather than particulate wax coats as brochosomes are more effective in protection from excrement and water. The adult abdomen, with more brochosomes showed higher hydrophobicity and lower pull-off forces than that of nymphs, and the CA values of abdomen were similar for both adults and nymphs on the bared (brochosome-free) integuments. These results demonstrate that it is brochosomes rather than microstructure that are chiefly responsible for hydrophobicity in this leafhopper.

Electron micrographs showed that brochosome density on the abdomen was higher in more mature nymphs and this, combined with their greater overall size and strength, accounted for their greater capacity to escape. During the development from nymph to adult, the distribution of microtrichia on ventral abdomen of *E. onukii* changed from nonuniform into uniform fish-scale array, which suggested they were well-covered by brochosomes. Such life-stage specific differences in adaptations to wetting are known for other insects. In water striders, for example, short microtrichia serve to waterproof the larvae whilst, in adults, this same function conferred by setae^30^.

Nymphs had difficulty removing their abdomen from the water droplets due to the strong adhesive force between the abdomen and water. We initially speculated that the adhesive force was derived from interaction between the cuticles of nymph abdomen and the water droplet. The surfaces of rose petals with micro- and nanoscale hierarchical structures have superhydrophobicity and a strong adhesive force, which was described as Cassie impregnating wetting status, and different to “lotus - effect”^31^. But we did not find micro- and nanoscale hierarchical structures on the cuticles of nymph abdomens, so the adhesive force is different from the force happen on the rose petals with water droplets. This is in apparent conflict with the high level of hydrophobicity evident in the contact angle test. We speculate that this is a scale effect and reflects the fact that the distribution of brochosomes on the nymph abdomen is nonuniform. When the nymph abdomen is in contact with a discrete and relatively small water droplets as in the contact angle study, the abdomen exhibits a hydrophobic state. In contrast, when fully immersed in a larger water volume, the fluid may adhere to areas of the abdomen and the area between appendages and abdomen where brochosomes coverage is less complete. The situation was different on *E. onukii* adults. Aside from this life stage being stronger, its abdomen had rough, uniform fish-scale structures formed by microtrichia, giving it a low pull-off force. In this respect, the abdomen microstructure of the leafhopper adults was similar to that of the dung beetle *Copris ochus* Motschulsky, which can freely go through the sticky faeces and soil without adhering to them due to pits and protrusions found on its head and body, minimizing adherence of water droplets or particles to body surface^32^,^33^.

Our study provides the first detailed understanding of the behavioural and microstructural adaptations of *E. onukii* to wetting and expands on earlier studies of other taxa of small pest insects such as aphids and planthoppers^8^,^34^. Further studies may lead to the development of improved biomimetic materials and structures. In the shorter term, the management of small pest insects can be enhanced by increased understanding of the biological adaptations to wetting and dislodgement. In Japan, Sato^35^ reports the use of a blower-type machine which sprays a strong wind mixed with small quantity of water to remove small pests including *E. onukii*. Applications of insecticides, or even overhead irrigation to dislodge pests, may be better timed to target the most vulnerable life stages. From above, improved surfactants that are particularly effective at wetting insects and reducing escape may increase the impact of pesticide sprays or – if cheap and environmentally benign – may be added to irrigation water.

## Materials and Methods

### Insect samples

*Empoasca onukii* individuals were collected from a tea plantation located on the campus of Fujian Agriculture and Forestry University (Fuzhou, Fujian Province, China, 119.2 E, 26.1°N). They were reared on fresh tea shoot cuttings that were standing in water under laboratory conditions (26 °C, RH 75~85%, 14 L:10D photoperiod) for at least two generations prior to the experiments.

### Escape behaviour

Adults and nymphs were first anesthetized with CO_2_ and individually placed on the abaxial surface of tea leaves. When they started to recover, the leaf was slowly moved into a vertical position reflecting its natural orientation with the shoot fixed on a block of moist floral foam. Insects were allowed to settle for 3 min in this position, which was a sufficient time for the insect to fully recover. For studies with adults, water was then sprayed with a hand sprayer (Yuyao Bluebird Garden Tools Co. Ltd., Ningbo, ZheJiang, China) until the insect escaped. This was recorded at 50 frames per second with a video camera (Sony 360E, Shanghai, China). The escape time was determined using Sony Vegas pro 11.0 software. For the nymphs, water was sprayed in the same manner for 1 min or until the insect was washed from the leaf. Nymphs remaining on the leaf were monitored for an additional 2 min to determine whether they were able to escape water droplets. Nymph behaviour was recorded as described above and escape time, when this occurred, was determined using Sony Vegas pro 11.0 software. The average volume of droplets was 0.2 ± 0.05 μL and the time for water droplets to travel from the sprayer nozzle to the leaf was 0.23 ± 0.07 s. Twenty replicates were used for each of the four categories of leafhoppers: N1, 1^st^ instar; N2-N3, 2^nd^ -3^rd^ instars; N4-N5, 4^th^ -5^th^ instars; and adults.

### Microstructure of pretarsi and abdomen

#### Microstructure of pretarsi

Eight adults and eight nymphs of late instars (N4-5) were dissected under 40× magnification (Olympus SZX16, Shanghai, China) after fixation with 2.5% glutaraldehyde for 24 h at 4 °C. The insects were then rinsed four times for 15 min in phosphate buffer saline (PBS, 0.1 M, pH 7.2) and then dehydrated in a graded ethanol series^16^,^36^. The samples were air-dried and mounted on copper stubs with double-sided copper sticky tape and sputtered with 25 nm gold/palladium (40/60) in a KEOLLV 1680 (EIKO, Japan) high resolution sputter coater. The samples were subsequently examined using scanning electron microscopy (SEM) (Jeol JSM-6380 LV SEM) operated at 15 kV.

#### Microstructure of abdomen

Samples of 16 adults and 12 nymphs for each instar (N1-5) were prepared as described for pretarsi studies. Brochosomes were removed from half of the insects in each sample using polyvinylsiloxane (PVS) (3 M ESPE, Dental Products St. Paul, MN 55144, USA) following Rakitov and Gorb^15^ allowing a clearer view of the underlying microstructure of the integument.

### Properties of pretarsi and abdomen

#### Wettability of pretarsi

To examine the interactions between the leafhopper pretarsi and water surface, a Petri dish was filled with 15 mL of water or soybean oil (as a control for surface tension) and placed on the stage of a microscope (Olympus SZX16, Shanghai, China). An adult (n = 20/substrate) or a nymph of 4–5th instar (n = 20/substrate) was transferred onto the liquid surface using a brush. Wings were removed from the adults to prevent escape. The reaction of the insect to the liquid was recorded using a digital camera with macro lens (Nikon D60, Thailand). The surface tensions of the water and soybean oil were measured by surface tension apparatus (BZY-2, Shanghai Hengping Scientific Instrument Co. Ltd., Shanghai, China) at 25 °C and approximately 50% RH (results: 71.9 ± 0.61 dyne × cm^−1^ for water and 39.1 ± 1.67 dyne × cm^−1^ for oil).

#### Contact angle measurement of abdomen

To examine the wettability of the abdomen surface, we used the sessile drop method^3^ in which a droplet of water was placed on the abdomen and an optical contact angle (CA) meter (Automatic Contact Angle Meter, SL200A, Shanghai, China) measured the extent to which the droplet either beaded or flattened against the insect. First, insects were killed with ether and fixed on glass slides with double-sided adhesive tape with either the ventral or dorsal surface exposed. Because a CA measurement requires the substrate to have a flat surface, an entomological needle was used to pierce and flatten the abdomen, with paper tissue used to absorb body contents. Water droplet size was 1.5 μL for adults and 1 μL for nymphs (4-5th instar). Measurements were made for the dorsal and ventral surfaces of each insect and for insects with intact brochosomes and from which brochosomes were removed. Eight insects were used for each category and treatment.

#### Pull-off force of pretarsi from leaf surface and from water

A custom-made balance, based on Eisner^37^, was used to measure the force required to pull insects from the surface of a water droplet and thereby indicate the level of difficulty associated with their escape. This was compared with force required to pull insects from the surface of a tea leaf. A 12 cm long plastic drinking straw was mounted on a stand with an 8 cm long cotton thread attached to each end. One string was connected to a gold wire (∅10 μm × 3 cm) that was attached to the live insect’s thoracic notum using silver glue. Sections of gold wire (∅10 μm × 2 cm) were attached to the opposite as adjustable weights. We first added sufficient gold wire to keep the straw balanced against the mass of the insect (W1 = mass × g) without touching any surface. The insect was then placed on the abaxial surface of a tea leaf. To estimate the pull off force of the insect from the leaf, additional gold wire weights (W2) were added until the insect was pulled from the leaf. The pull-off force (POF) was estimated as the difference between W2 and W1. To measure POF from water, an insect was first weighed (W1) as previously described and then transferred onto a 6 μL water droplet located on the abaxial surface of a tea leaf. The POF from water was measured in a similar fashion by adding gold wires until the insect was pulled from the water droplet (W2). For both substrates, POF was measured for 8 nymphs (4-5th instar) and 8 adults. To obtain a direct measure of the mass of these small insects, the weight of three batches of 50 adults and 50 nymphs was determined using an electronic analytical balance (d = 0.1 mg) (Sartorius Scientific Instruments Co. Ltd, Beijing).

#### Pull-off force of abdomen from water

The POF was used to evaluate the adhesive force between the insect abdomen and a water droplet as an indicator of the hydrophobicity of the abdomen. As the leafhopper hind legs of both nymphs and adults cover the ventral abdomen, the insects were anesthetized by exposing them to a CO_2_ atmosphere for 30 s, the hind legs were removed and dental wax of low melting temperature was applied to seal the wounds. The POF method was as described for pretarsi except the sample insect was transferred onto the 3 μL water droplet with only its abdomen touching the water surface. Four groups were tested: adults, nymphs (4-5th instar) and each of them following brochosome removal (n = 8 per group).

## Additional Information

**How to cite this article**: Lin, M. *et al.* Avoidance, escape and microstructural adaptations of the tea green leafhopper to water droplets. *Sci. Rep.* **6**, 37026; doi: 10.1038/srep37026 (2016).

**Publisher’s note**: Springer Nature remains neutral with regard to jurisdictional claims in published maps and institutional affiliations.

## Supplementary Material

Supplementary Information

Supplementary Video 1

Supplementary Video 2

Supplementary Video 3

## Figures and Tables

**Table 1:** Escape behaviour of four life stages of *Empoasca onukii* in response to water spraying.

Stage	Behaviour/outcome	Proportional rate of successful escape (n = 20)	Mean escape time of escaped individuals (s)
N1	All trapped in the water and rapidly washed away	0	—
N2~N3	Most trapped in water droplets and able to crawl from droplets only slowly after cessation of spraying	0.15	48.7 ± 3.05
N4~N5	Minority trapped in water droplets and able to crawl from droplets relatively rapidly after cessation of spraying	0.85	17.4 ± 2.78
Adult	Jumping away during spraying	1.0	0.26 ± 0.02

Water was sprayed for 1 minute to adults and immatures of various nymphal (N) stages. The proportional rate of successful escape was calculated in the 2 minutes after the cessation of spraying for 1 min.

## References

[b1] Cong Q., Chen S. C., Fang Y. & Ren L. Q. Super-hydrophobic characteristics of butterfly wing surface. J. Bionic. Eng. 1, 249–255 (2004).

[b2] Fang Y., Sun G. & Wang T. Q. Effect of non-smooth scale on surface wettability of butterfly wing. Chinese Sci. Bull. 52, 354–357 (2007).

[b3] Sun M. X., Watson G. S., Zheng Y. M., Watson J. A. & Liang A. P. Wetting properties on nanostructured surfaces of cicada wings. J. Exp. Biol. 212, 3148–3155 (2009). 19749108 10.1242/jeb.033373

[b4] Gao X. F. & Jiang L. Water-repellent legs of water striders. Nature 432, 36 (2004). 15525973 10.1038/432036a

[b5] Mann J. A., Iatchell G. M., Dupuch M. J., Harrington R., Clark S. J. & McCartney H. A. Movement of apterous *Sitobion avenae* (Homoptera: Aphididae) in response to leaf disturbances caused by wind and rain. Ann. Appl. Biol. 126, 417–427 (1995).

[b6] Li R. B. A new method to control pink tea rust mite *Acaphylla theae* Watt by water spraying. Tea Comm. 2, 12 (1996).

[b7] Liang H. B., Zhang R. Z., Zhuang G. X., Wen Z. L. & Wang G. P. Infestation levels of *Diuraphis Noxia* Mordvilko response to precipitation and irrigation. Acta Ent. Sin. 41, 382–388 (1998).

[b8] Wang B., Li K. B., Yin J. & Cao Y. Z. Effects of simulated wind and rain on the natural population dynamics of *Macrosiphum avenae. Chinese* J. Appl. Entomol. 48, 1646–1654 (2011).

[b9] Narayandas G. K. & Alyokhin A. V. Interplant movement of potato aphid (Homoptera: Aphididae) in response to environmental stimuli. Environ. Entomol. 35, 733–739 (2006).

[b10] Watson G. S., Cribb B. W. & Watson J. A. Contrasting micro/Nano architecture on termite wings: ttwo divergent strategies for optimising success of colonisation flights. PLoS One 6 **(9)**, e24368 (2011). 21935401 10.1371/journal.pone.0024368 PMC3173396

[b11] Watson G. S., Cribb B. W. & Watson J. A. The role of micro/nano channel structuring in repelling water on cuticle arrays of the lacewing. J. Struct. Biol. 171, 44–51 (2010). 20347993 10.1016/j.jsb.2010.03.008

[b12] Rakitov R. A. Brochosomal coatings of the integument of leafhoppers (Hemiptera, Cicadellidae). In Functional Surfaces in Biology (ed. Gorb S. N.), 113–137 (Springer Netherlands, 2009).

[b13] Rakitov R. A. Secretion of brochosomes during the ontogenesis of a leafhopper, *Oncometopia orbona*(F.) (Insecta, Homoptera, Cicadellidae). Tissue Cell 32, 28–39 (2000). 10798315 10.1054/tice.1999.0084

[b14] Rakitov R. A. What are brochosomes for? An enigma of leafhoppers (Hemiptera, Cicadellidae). Denisia 4, 411–432 (2002).

[b15] Rakitov R. A. & Gorb S. N. Brochosomal coats turn leafhopper (Insecta, Hemiptera, Cicadellidae) integument to superhydrophobic state. Proc. R. Soc. B. 280, 1752 (2013)a. 10.1098/rspb.2012.2391 PMC3574307 23235705

[b16] Dong D. Y. & Huang M. Analysis of the Anointing and Grooming Behavior of Several Adult Insects in Typhlocybinae (Hemiptera: Cicadellidae). J. Insect Behav. 26, 540–549 (2013).

[b17] Rakitov R. A. & Gorb S. N. Brochosomes protect leafhoppers (Insecta, Hemiptera, Cicadellidae) from sticky exudates. J. R. Soc. Interface 10, 445 (2013)b. 10.1098/rsif.2013.0445 PMC3758005 23904586

[b18] Qin D. Z., Zhang L., Xiao Q., Dietrich C. & Matsumura M. Clarification of the identity of the tea green leafhopper based on morphological comparison between Chinese and Japanese specimens. PloS One. 10, e0139202 (2015). 26422616 10.1371/journal.pone.0139202 PMC4589377

[b19] Zhu J. Q. Studies on numerical prediction of *Empoasca vitis* (Göthe) at the first population peak. Acta Agi. Zhejiangensis 3, 195–197 (1991).

[b20] Li H. L. & Lin N. Q. The influence of temperature and humidity on the population dynamics of small green leafhopper at tea garden. Fujian J. Agr. Sci. 27, 55–59 (2012).

[b21] Wang R. Z. & Huang P. Y. Biological observation of the tea green leafhopper. Jiangxi Plant Prot. 23, 105–106 (2000).

[b22] Pu X. Y. & Feng M. G. Efficacy of emulsifiable formulations of two entomopathogenic fungi against small green leafhoppers on tea plant. Chinese. J. Appl. Ecol. 15, 619–622 (2004). 15334957

[b23] Pu X. Y., Feng M. G. & Shi C. H. Impact of three application methods on the field efficacy of a Beauveria bassiana-based mycoinsecticide against the false-eye leafhopper, *Empoasca vitis* (Homoptera: Cicadellidae) in the tea canopy. Crop Prot. 24, 167–175 (2005).

[b24] Snodgrass R. E., Eickwort & George E. The thoracic legs in Principles of insect morphology. 167 (Cornell University Press, 1993).

[b25] Brackenbury J. Targetting & optomotor space in the leaf-hopper *Empoasca vitis* (Göthe) (Hemiptera: Cicadellidae). J. Exp. Biol. 199, 731–740 (1996). 9318490 10.1242/jeb.199.3.731

[b26] Lee Y. I., Kogan M. & Larsen J. R. Jr. Attachment of the potato leafhopper to soybean plant surfaces as affected by morphologyof the pretarsus. Entomol. Exp. Appl. 42, 101–107 (1986).

[b27] Losey J. E. & Denno R. F. Positive predator-predator interactions: enhanced predation rates and synergistic suppression of aphid populations. Ecology 79, 2143–2152 (1998).

[b28] Hu H. M., Watson G. S., Cribb B. W. & Watson J. A. Non-wetting wings and legs of the cranefly aided by fine structures of the cuticle. J. Exp. Biol. 214, 915–920 (2011). 21346118 10.1242/jeb.051128

[b29] Dill L. M., Fraser A. H. G. & Roitberg B. G. The economics of escape behaviour in the pea aphid, Acyrthosiphon pisum. Oecologia 83, 473–478 (1990). 10.1007/BF00317197 28313180

[b30] Goodwyn P. J. P., Voigt D. & Fujisaki K. Skating and diving: changes in functional morphology of the setal and microtrichial cover during ontogenesis in *Aquarius paludum* Fabricius (Heteroptera, Gerridae). J. Morphol. 269, 734–744 (2008). 18302188 10.1002/jmor.10619

[b31] Feng L., Zhan Y. A. g, Xi J. M., Zhu Y., Wang N., Xia F. & Jiang L. Petal effect: A superhydrophobic state with high adhesive force. Langmuir 24, 4114–4119 (2008). 18312016 10.1021/la703821h

[b32] Ren L. Q., Wang Y. P., Li J. Q. & Tong J. Flexible unsmoothed cuticles of soil animals and their characteristics of reducing adhesion and resistance. Chinese Sci. Bull. 43, 166–169 (1998).

[b33] Heng H., Sun J. R., Li J. Q. & Ren L. Q. Structure of the integumentary surface of the dung beetle *Copris ochus* Motschulsky and its relation to non-adherence of substrate particles. Acta Bioph. Sin. 17, 785–792 (2001).

[b34] Sato Y. Integrated tea pest management in Japan. International tea symposium, Hangzhou, China. 280–282 (2014).

[b35] Ma M. Y., Wu S. W. & Peng Z. P. Population Seasonality: Will They Stay or Will They Go? A Case Study of the *Sogatella furcifera* (Hemiptera: Delphacidae). J Insect Sci. 15, 61–66 (2015). 26009632 10.1093/jisesa/iev040 PMC4535475

[b36] van Baarene J., Barbier R. & Nénon J. P. Females antennal sensilla of *Epidinocarsis lopezi* and *Leptomastix dactylopii*(Hymenoptera:Encyrtidae), parasitoids of pseudococcid mealybugs. Can. J. Zool. 74, 710–720 (1996).

[b37] Eisner T. Tales from the Website in For love of insects (ed Eisner T.) 207–209. (The Belknap Press, 2005).

