# Supplementary material for: Avoidance, escape and microstructural adaptations of the tea green leafhopper to water droplets
Source: Sci Rep. 2016 Nov 15;6:37026. doi: 10.1038/srep37026 (PMC5109466; doi:10.1038/srep37026)
Supplement: Supplementary Information [file srep37026-s1.doc]

**Avoidance, escape and microstructural adaptations** **of the tea green leafhopper to water droplets**

**Meizhen Lin ^a,b,c^, Liette Vasseur ^a,d^, Guang Yang ^a,b,c^, Geoff M. Gurr^a,b,e^ and**

**Minsheng You ^a,b,c *^**

**Supplementary Information**

Additional Supporting information may be found in the online version of this article:

**Video S1.** Escape behavior of adult ***E. onukii*** when stimulated by water spraying.

**Video S2.** Escape behavior of the younger nymph of ***E. onukii*** when stimulated by water spraying.

**Video S3.** Escape behavior of the older nymph of ***E. onukii*** when stimulated by water spraying.
